# Diet Transition from High-Forage to High-Concentrate Alters Rumen Bacterial Community Composition, Epithelial Transcriptomes and Ruminal Fermentation Parameters in Dairy Cows

**DOI:** 10.3390/ani11030838

**Published:** 2021-03-16

**Authors:** Sonny C. Ramos, Chang Dae Jeong, Lovelia L. Mamuad, Seon Ho Kim, Seung Ha Kang, Eun Tae Kim, Yong Il Cho, Sung Sill Lee, Sang Suk Lee

**Affiliations:** 1 Ruminant Nutrition and Anaerobe Laboratory, Department of Animal Science and Technology, Sunchon National University, Suncheon 57922, Korea; ynnosomarc@yahoo.com.ph (S.C.R.); cdvf12@hanmail.net (C.D.J.); loveliamamuad2306@gmail.com (L.L.M.); mhs0425@hanmail.net (S.H.K.); 2 The University of Queensland Diamantina Institute, Faculty of Medicine, The University of Queensland, Brisbane, QLD 4072, Australia; kansbio@gmail.com; 3 Dairy Science Division, National Institute of Animal Science, Rural Development Administration, Cheonan 31000, Korea; etkim77@korea.kr; 4 Animal Disease and Diagnostic Laboratory, Department of Animal Science and Technology, Sunchon National University, Suncheon 57922, Korea; ycho@scnu.ac.kr; 5 Institute of Agriculture and Life Science and University-Centered Labs, Gyeongsang National University, Jinju 52828, Korea; lss@gnu.ac.kr

**Keywords:** dairy cows, changing diet, rumen fermentation, bacterial community, transcriptome

## Abstract

**Simple Summary:**

Cattle are fed a high-concentrate diet to improve their productivity; however, it alters the rumen ecosystem due to high structural carbohydrates level, resulting in ruminal acidosis. This study investigated the effect of changing diet on ruminal fermentation parameters, bacterial community composition, and expressed genes of Holstein Friesian cows, with changes induced by transition from a high-forage to two succeeding high-concentrate diets, and then returned to a high-forage diet. Ruminal pH drastically decreased; however, ammonia nitrogen, and individual and total volatile fatty acid (VFA) concentrations increased during the high-concentrate diet period. High-concentrate diet also reduced rumen bacterial richness and diversity. Gene expression in rumen epithelia was affected and altered by changing diet through the obtained differentially expressed genes.

**Abstract:**

Effects of changing diet on rumen fermentation parameters, bacterial community composition, and transcriptome profiles were determined in three rumen-cannulated Holstein Friesian cows using a 3 × 4 cross-over design. Treatments include HF-1 (first high-forage diet), HC-1 (first high-concentrate diet), HC-2 (succeeding high-concentrate diet), and HF-2 (second high-forage diet as a recovery period). Animal diets contained Klein grass and concentrate at ratios of 8:2, 2:8, 2:8, and 8:2 (two weeks each), respectively. Ammonia-nitrogen and individual and total volatile fatty acid concentrations were increased significantly during HC-1 and HC-2. Rumen species richness significantly increased for HF-1 and HF-2. Bacteroidetes were dominant for all treatments, while phylum Firmicutes significantly increased during the HC period. *Prevotella*, *Erysipelothrix*, and *Galbibacter* significantly differed between HF and HC diet periods. *Ruminococcus* abundance was lower during HF feeding and tended to increase during successive HC feeding periods. *Prevotella* *ruminicola* was the predominant species for all diets. The RNA sequence analysis revealed the keratin gene as differentially expressed during the HF diet, while carbonic-anhydrase I and S100 calcium-binding protein were expressed in the HC diet. Most of these genes were highly expressed for HC-1 and HC-2. These results suggested that ruminal bacterial community composition, transcriptome profile, and rumen fermentation characteristics were altered by the diet transitions in dairy cows.

## 1. Introduction

Dairy cattle feeding patterns have been changed to provide the required energy and nutrients by feeding them concentrate feeds instead of fiber-rich forages [1]. Dairy cattle frequently undergo dietary transitions to meet the energy requirements for milk production around the start of their lactation period [2]. Such dietary transitions have supported the increase in milk yields; however, they raise concerns about rumen function in these cattle [1,3,4]. These dietary transitions affect chewing behavior and rumen buffering, which may lead to accumulation of large amounts of volatile fatty acids (VFA) in the rumen fluid [5]. Furthermore, the transition of the diet from high forage to high grain results in greater VFA concentrations and accumulation of lactate in the rumen, thus increasing the risk of subacute ruminal acidosis (SARA) [6,7,8,9,10]. SARA has been associated with metabolic and microbial alterations, and imbalances in the rumen, which are involved in metabolic health disorders in dairy cattle [11,12]. Among its various manifestations, it reduces cellulolytic bacteria counts, leading to impairment of bacterial activity due to undesirable ruminal pH, which may result in low fiber degradation [13,14,15,16]. Energy and essential nutrients are mainly obtained by ruminants through a complex symbiotic relationship with the rumen microbiome [17]. The health and efficiency of the host ruminants are significantly affected by changes in the bacterial community in the rumen [18]. While a high-forage diet is usually switched to a high-concentrate diet to improve the productivity of ruminants, it can alter the rumen ecosystem [17].

The rumen is a complicated environment of microbes and hosts numerous amounts of bacteria that constitute an efficient mutual relationship of host animal and microorganisms [19,20,21]. Ruminants rely on ruminal microbes for the degradation of structural carbohydrates, and VFA and microbial protein synthesis served as the main sources of protein and energy [22]. The combined activities of a wide range of bacterial community in the rumen metabolizes the carbohydrates from feed [23]. Normally, various groups of bacteria are used to specialize for the utilization of specific types of polysaccharides, such as starch or cellulose, which are known to be digested by saccharolytic and cellulolytic bacteria, respectively [24]. Starch is one of the most abundant polysaccharides found in high-grain diets, which are mainly used to increase animal performance and are more favorable to saccharolytic bacteria [25,26,27,28]. However, the use of starch-rich feedstuffs was associated with the reduction of bacterial richness and diversity in the rumen and large intestines [23], which resulted in a decrease in relative abundance of Bacteroidetes and an increase in case of Firmicutes [29,30,31]. Ruminal saccharolytic bacteria can be considered as potential initiators of SARA, as higher starch basically results in greater production rates of short-chain fatty acids [23]. A previous study investigated the impact of SARA on the rumen microbial community using high-throughput 16S rRNA sequencing and found that there was a major shift in the three dominating phyla [1]. Another next-generation sequencing (NGS) technique is the RNA sequencing technology, which is an indispensable tool for meta-transcriptome analysis of different gene expression [32]. Meta-transcriptome analysis is more powerful compared to other NGS methods because of the information it can provide about microbial populations that are transcriptionally active [33], as well as the in-depth analysis of functional gene activity and metabolic pathways [34]. However, meta-transcriptome analysis in rumen with regards to SARA is limited [35]. In addition, despite adaptation and recovery of rumen bacteria during SARA challenge through high grain-feeding, the effect of changing diet in dairy cattle is largely unclear. This study was, therefore, undertaken to investigate the effect of changing diet on ruminal fermentation parameters, bacterial community composition, and transcriptome profile of Holstein Friesian cows, with changes induced by transition from a high-forage diet to two succeeding high-concentrate diets and then back to a high-forage diet. The findings provide plausible information that diet transitions would induce changes in ruminal pH and fermentation ability, consequently altering rumen bacterial community composition and transcriptome profile in dairy cows.

## 2. Materials and Methods

### 2.1. Animal Care

This study was conducted at the Sunchon National University (SCNU) animal farm and in the Ruminant Nutrition and Anaerobe Laboratory, the Department of Animal Science and Technology, SCNU, Suncheon, Jeonnam, Korea. The animals used and all experimental protocols were reviewed and approved by the Sunchon National University Animal Research Ethics Committee (SCNU IACUC, approval number: SCNU IACUC-2018-01).

### 2.2. Animals, Feeding, and Experimental Design

Three rumen-cannulated Holstein Friesian cows, with average body weights of 600 ± 47 kg, were used in this study. A 3 × 4 cross-over design was used for this experiment to evaluate the effects of changing diet on rumen fermentation, bacterial composition, and transcriptome profile of the animals. This changing diet challenge was defined as the high-forage period followed by two succeeding high-concentrate periods, and then a return to the high-forage period to investigate the changes in ruminal pH and the adaptive capability of the rumen microbiome. Prior to the experiment, cows were fed with mixed Klein grass hay and concentrate at a ratio (kg) of 8:2. During the experimental period, all cows were fed twice a day continuously with the same diet ratio for 2 weeks. Then, after the first period, diet was changed to the ratios (forage:concentrate) as follow: 2:8 (2 weeks), 2:8 (2 weeks), and 8:2 (2 weeks). These feeding ratios served as treatments (HF-1 (first high-forage diet), HC-1 (first high-concentrate diet), HC-2 (succeeding high-concentrate diet, and HF-2 (second high-forage diet, served as a recovery period)). The HF-1 consisted of dairy cows fed a high-forage diet for 2 weeks. Then, it was followed by HC-1, which involved the same group of cows fed for 2 weeks with high-concentrate right after the HF-1 period prior to acidosis. Afterwards, a succeeding 2 weeks of feeding with high-concentrate diet were allotted for HC-2 in the same group of cows, followed by a transition to a high-forage diet (HF-2), which served as a recovery period for the animals. Animals were housed in individual stalls and had free access to water. The concentrate given to dairy cows was supplied by Purina^®^ Cargill, Korea. The chemical compositions of the forage and concentrate fed to the dairy cows are shown in Table 1.

### 2.3. Rumen Fluid Collection and Analysis of Ruminal Fermentation Parameters

Ruminal contents were collected from the three rumen-cannulated cows before morning feeding on the last day of each period before transitioning to the next diet period. The fluid samples were squeezed and strained through a four-layer surgical gauze and placed in an amber bottle, which was subsequently capped after collection. The collected fluid samples were maintained at 39 °C and immediately transported to the laboratory for analysis of the ruminal fermentation parameters. An aliquot of rumen fermenta was separated from the sample bottles, transferred to two 1.5 mL microcentrifuge tubes, and stored at −80 °C, prior to ammonia-nitrogen (NH_3_-N) and VFA analyses. Following this, the samples were thawed at room temperature and were centrifuged for 10 min at 13,000 rpm at 4 °C using a Micro 17TR centrifuge (Hanil Science Industrial, Korea). The obtained supernatant was used for NH_3_-N and VFA concentration analyses. The NH_3_-N concentration was measured according to the colorimetric method developed by Chaney and Marbach [36] using a Libra S22 spectrophotometer (Biochrom Ltd., CB40FJ, Cambridge, UK) at an absorbance of 630 nm. The analysis of the VFA concentration was done using high-performance liquid chromatography (Agilent Technologies 1200 series, Tokyo, Japan) with an ultraviolet (UV) detector set at 210 and 220 nm. Samples were isocratically eluted with 0.0085N H_2_SO_4_ at a flow rate of 0.6 mL/min and a column temperature of 35 °C [37].

### 2.4. Metataxonomic and Transcriptomics Analyses

Rumen fluid samples for metataxonomic analysis and rumen tissue biopsies for transcriptomics analysis were obtained from each treatment, and were sent to Macrogen (Seoul, Korea) for sample preparation, library construction, sequencing, and data analysis.

For metataxonomic analysis, after quality assessment of extracted DNA from the samples, a survey of bacterial community composition was performed. Libraries were constructed by targeting the V3–V4 variable regions of the 16S ribosomal RNA (rRNA) gene using Herculase II Fusion DNA Polymerase Nextera XT Index Kit V2 following the Illumina’s 16S Metagenomic sequencing library preparation. After assessing the quality of the constructed libraries, sequencing was done using the Illumina sequencing platform (Miseq). Then, the generated paired-end (PE) sequencing raw reads from the base call binary data obtained by real-time analysis were converted to fastq format. Filtering of PE raw reads from overhang adapter sequences were done, then the filtered PE reads were merged. Quality filtering, trimming of short and extra-long reads, and removal of duplicate reads were done, then the filtered reads were clustered at 100% identity using CD-HIT-OTU [38]. After identifying chimeric reads, noise filtering was done, and the remaining clusters were binned to operational taxonomic units (OTU) with a cut-off value of 97% species level identity using the same program. Afterwards, taxonomic assignment of representative sequences from observed OTUs were matched by BLAST on the NCBI 16S rRNA database using Quantitative Insights Into Microbial Ecology (QIIME-UCLUST) [39]. The bacterial diversity was graphically presented using Metagenomics Core Microbiome Exploration Tool (MetaCOMET) [40] by uploading a biological observation matrix to the web server [41], generated using Mothur [42].

For transcriptomics analysis of the host’s response to challenge diet, total RNA were extracted from the rumen epithelial tissue biopsy. DNA contamination was eliminated using DNAse. After quality assessment of extracted RNA from the samples, libraries were prepared using the TruSeq Stranded Total RNA LT Sample Prep Kit (Illumina, San Diego, CA, USA) with Ribo-Zero following the manufacturer’s Prep Guide. Sequencing was carried out using the TruSeq 3000 4000 SBS Kit v3 in an Illumina HiSeq 4000 sequencer (Illumina, San Diego, CA, USA). The sequenced raw reads were subjected to quality control by calculating the overall reads’ quality, total bases, total reads, and guanine-cytosine (GC) percentage. Then, sequencing artifacts like low-quality reads and adapter sequences were filtered out using Trimmomatic [43]. The remaining trimmed reads were then mapped against cow reference genome (Bos_taurus_UMD_3.1.1/bosTau8) retrieved from the University of California Santa Cruz genome database website (genome.ucsc.edu), using hierarchical indexing for spliced alignment of transcripts 2 (HISAT2) [44] by handling spliced reads’ mapping using Bowtie2 aligner [45]. Transcripts were assembled using StringTie [46] with aligned reads to provide information of known, novel, and alternative splicing transcripts based on the reference genome model. Afterwards, the level of abundance of transcripts in fragments per kilobase of transcript per million mapped reads (FPKM) was normalized. The FPKM value of known genes was used to sort the differentially expressed genes among samples. Three comparison pairs were conducted, where sample HF-1 served as the point of comparison of the three samples to determine whether a certain known gene was up- or down-regulated between diet types. During this analysis, low-quality transcripts were filtered, then the FPKM normalization was performed. Statistical analysis was performed using Fold Change per comparison pair (fc), and significant genes were selected if the fold change per comparison pair was greater than or equal to two. Afterwards, hierarchical clustering analysis was performed among the significant genes in order to group the genes and samples based on similarity of expression patterns.

### 2.5. Statistical Analysis

All analyses were carried out using Statistical Analysis Systems (SAS) software version 9.4 (SAS Institute 2012) (SAS Institute Inc., Cary, NC, USA). Data were statistically evaluated using Proc Glimmix for a complete randomized design. The experiment was done twice, and the treatments were conducted in triplicate. Least square means was used to identify differences among treatments. A *p* < 0.05 was considered indicative of significant differences.

## 3. Results

### 3.1. The Effects of Treatments on Rumen Fermentation Parameters

The ruminal pH was significantly higher (*p* = 0.001) during the high-forage diets. As the feeding switched to a high-concentrate diet, a lower ruminal pH was observed. The rumen pH of HC-1 and HC-2 was significantly (*p* = 0.001) lower than the HF periods due to the high concentrate ratio in the diet. The NH_3_-N concentrations for HF-1 and HF-2 were significantly lower (*p* = 0.001) and had an opposite result to the cows receiving the high-concentrate diet. Furthermore, in the HC diet group, the individual and total VFA concentrations were significantly higher (*p* = 0.001) than the HF group. The acetate to propionate ratio was significantly lower (*p* = 0.001) during HC-1 and HC-2, then during the HF feeding period, an opposite result was observed. Meanwhile, a significantly lower (*p* = 0.001) VFA concentration was observed during the HF diet periods (Table 2).

### 3.2. General Ruminal Bacterial Community Composition

**Figure 1:**
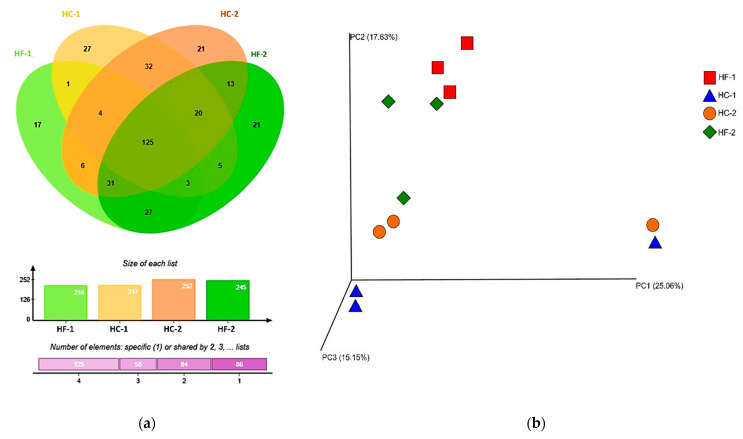
Venn diagram of rumen microbiome after transition of diet from high forage (HF-1) to high concentrate (HC-1 to HC-2), and back to high forage (HF-2), representing the unique, shared, and core microbiome. The bar graph below shows the size of representative species of observed operational taxonomic units (OTUs) per treatment. (**a**) Venn diagram was generated using jVenn [47]. (**b**) Principal coordinate analysis (PCoA) of all samples using Bray-Curtis distance derived from the subset of identified OTUs. EMPeror [48] was used to generate the PCoA plot. HF-1, high-forage diet; HC-1, high-concentrate diet; HC-2, high-concentrate diet; HF-2, high-forage diet.

The bacterial community of the rumen microbiome after diet transition is presented as a Venn diagram in Figure 1a. Out of 353 overall representative species of observed OTUs, a total of 125 (35.4%) were present across all communities (core), 142 (40.23%) were observed as shared by 2 or 3 communities, and a total of 86 (24.36%) were distributed uniquely in the four communities. This figure also shows that as the diet changes from high forage to high concentrate, the number of bacterial species also increases. However, at the point when the diet was reverted back to high forage (HF-2), the number of bacterial species decreased, but was still of higher count compared to HF-1. It can also be noted that these bacteria uniquely shared between two communities were higher between the same diet (HF-1 and HF-2: 27, HC-1 and HC-2: 32) compared to shared species between different diet types (HF-1 and HC-1: 1, HF-1 and HC-2: 6, HC-1 and HF-2: 5), except between HC-2 and HF-2, for which the number of species observed was higher than ten. The unweighted unifrac diversity principal coordinate analysis (PCoA) plots showed a similarity within the diet changing stage (Figure 1b). The high-forage diet periods (HF-1 and HF-2) had similar bacterial communities with non-distinct clusters and no spatial separation among populations. A portion of the bacterial community of HC-2 was also related to HF-2 due to the transition of the diet from high-concentrate to high-forage. There was also a tendency toward more similar bacterial communities between the high-concentrate diet periods (HC-1 and HC-2).

**Figure 2:**
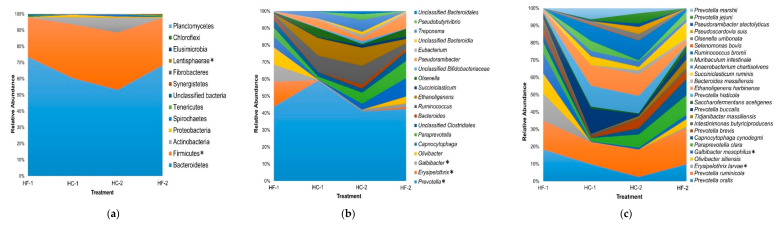
Relative abundance of the observed (**a**) phyla, (**b**) genera, and (**c**) species from the four different treatments. Relative abundance was computed using Quantitative Insights Into Microbial Ecology (QIIME) [39]. HF-1, high-forage diet; HC-1, high-concentrate diet; HC-2, high-concentrate diet; HF-2, high-forage diet. Asterisk (*) denotes significant differences (*p* < 0.05).

A total of 15 bacterial phyla were identified within the rumen bacteria communities. The most abundant phyla included Bacteroidetes, Firmicutes, and Actinobacteria. With these phyla, Bacteroidetes was dominant in all treatments. However, its relative abundance decreased as cattle received a high-concentrate diet and a notable increase in abundance when the diet changed to high forage. This was contrary to the relative abundances of Firmicutes. As the rumen was subjected to the high-concentrate diet condition, the abundance of Firmicutes significantly increased (*p* = 0.039). A similar response was observed with the phylum Actinobacteria. Its abundance increased as the high-concentrate diet condition was prolonged (Figure 2a).

The analysis of genus level composition revealed 143 identified genera, of which 19 were dominant (>1% of the relative abundance) (Figure 2b). The relative abundance of *Prevotella, Erysipelothrix*, and *Galbibacter* significantly differed between the high-forage and the high-concentrate diet periods (*p* < 0.05). *Prevotella* had a higher relative abundance during the first high-concentrate diet period. However, it significantly decreased (*p* = 0.024) as the high-concentrate diet was continuously supplied to cows. Meanwhile, *Erysipelothrix* and *Galbibacter* both showed similar responses to the treatments. These genera significantly decreased their abundances as the diet transitioned from high forage to high concentrate (*p* < 0.05), and gradually increased as the rumen recovered with the return to the high-forage diet. *Ruminococcus* had lower abundances under the high-forage diet period and tended to increase in abundance, though not significantly, during the two subsequent high-concentrate diet periods (*p* = 0.086). A similar pattern as with *Ruminococcus* was observed in the case of *Ethanoligenes* and *Treponema*, which showed high abundance during the periods of high-concentrate diet and the least in both high-forage diets.

At the species level, *Prevotella ruminicola* was the most dominant species for all treatments, with relative abundances of 12.61%, 9.57%, 11.70%, and 14.67%, respectively (Figure 2c). Its abundance decreased as the diet transitioned from the high-forage diet to the high-concentrate diet. Meanwhile, as the high-concentrate diet period continued, a notable increase in abundance was observed. A similar scenario was observed with *Paraprevotella clara, Prevotella brevis*, and *Capnocytophaga cynodegmi*. The transition of the diet from high forage to high concentrate increased the abundances of *Ethanoligenens harbinense, Ruminococcus bromii, Prevotella histicola, Prevotella jejuni, Olsenella umbonata, Anaerobacterium chartisolvens*, and *Pseudoramibacter alactolyticus.* There was also a continual increase in their relative abundances when the high-concentrate diet was supplied consecutively. Furthermore, *Prevotella oralis* and *Olivibacter sitiensis* had a drastic reduction of abundance after changing the diet from a high-forage to a high-concentrate diet; however, these particular species recovered when the diet returned back to a high-forage one. In case of *Prevotella buccalis* and *Prevotella marshii*, the contrary was observed when the diet was shifted from high-forage to high-concentrate. The relative abundance radically increased, but as the high-concentrate diet continued, a remarkable decrease in abundance was noticed. A statistical comparison of single species showed a significant effect of treatments only in the case of *Galbibacter mesophilus* and *Erysipelothrix larvae*. These species were both significantly more abundant for treatments with the high-forage diet (*p* = 0.006; *p* = 0.010). A heatmap was also generated showing the relationship of each bacterial species on the pH values of treatments clustered based on Pearson correlation (Figure S1). 

**Figure 3:**
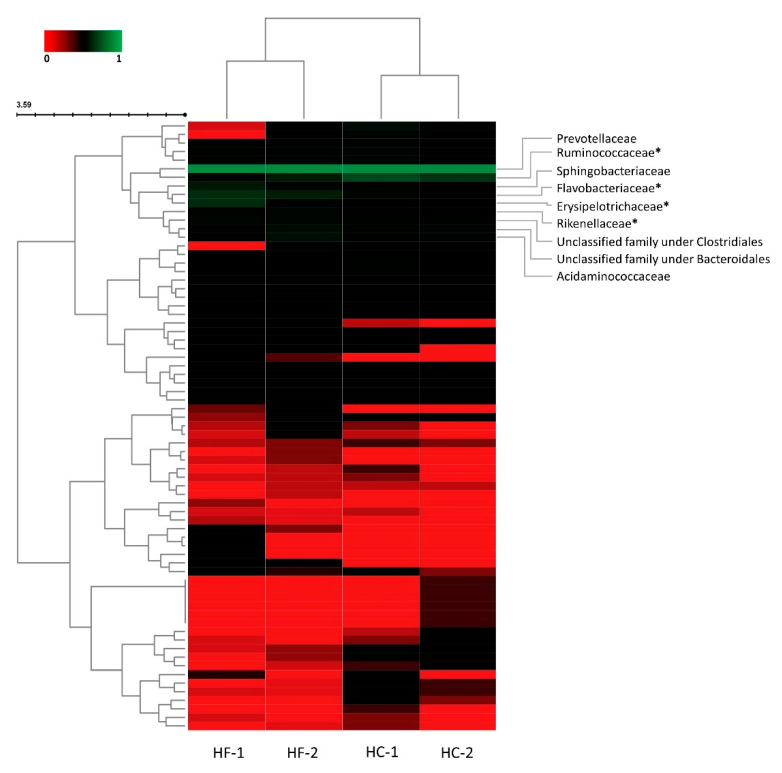
Heatmap clustering of observed families (rows) based on normalized value (0–1) of relative abundance per treatment (columns) using Bray-Curtis dissimilarity test and Ward linkage. Figure generated using Interactive Cluster Heatmap library (InCHlib) [49]. HF-1, high-forage diet; HC-1, high-concentrate diet; HC-2, high-concentrate diet; HF-2, high-forage diet. Asterisk (*) denotes significant differences (*p* < 0.05).

The normalized data presented in Figure 3 shows the clustering based on similarity of relative abundance of families and treatments. The four communities after the diet transitions were clustered based on diet type, indicating close similarity of family abundance in each diet type. On the other hand, families were clustered based on their relative abundance, distinguishable by color based on their normalized value, by which two major clusters were formed: cluster of families with low relative abundance (lower cluster), and families with higher relative abundance (upper cluster). Distinctly observed is a subcluster containing the highest relative abundance, and among the families in this cluster, Ruminococcaceae, Flavobacteriaceae, Erysiopelotrichaceae, and Rikenellaceae displayed significant differences (*p* < 0.05) between treatments. Family Ruminococcaceae was initially lower during the HF diet, but significantly increased as the diet was altered to HC, and dropped when the diet was reverted to HF. This pattern was oppositely observed in Family Flavobacteriaceae, wherein abundance had decreased when changed to HC, then increased significantly when diet was reverted to HF. At the same time, Families Erysipelotrichaceae and Rikenellaceae were observed to be higher in HF1 and HF2, respectively.

**Figure 4:**
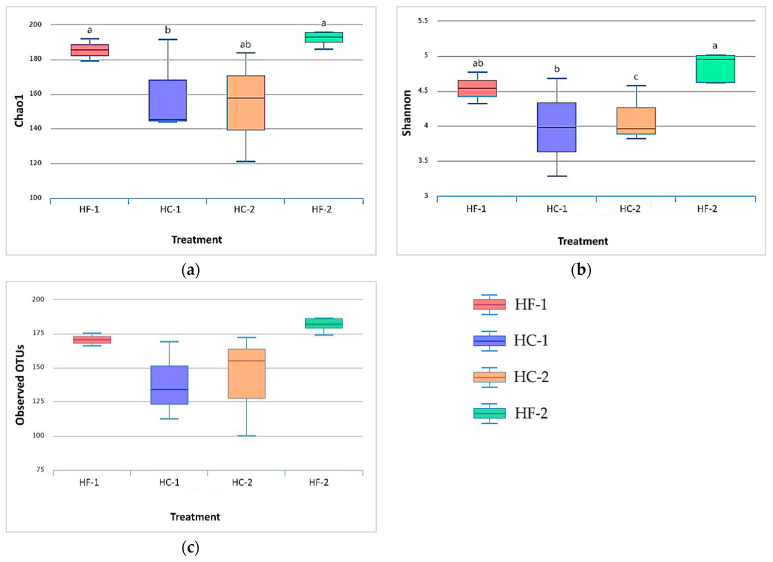
Boxplot representation of alpha diversity indices: (**a**) chao1, (**b**) Shannon, and (**c**) observed OTUs, between diet groups. Alpha-diversity metrics’ visualization were done in MetaCOMET [40] and computed using QIIME [39]. HF-1, high-forage diet; HC-1, high-concentrate diet; HC-2, high-concentrate diet; HF-2, high-forage diet. ^a–c^ superscript denotes significant differences (*p* < 0.05).

The boxplot representation of alpha diversity indices is shown in Figure 4. Alpha diversity indices are composite indices that reflect abundance and consistency. The Chao1, which estimates species richness, significantly increased during the high-forage diet (*p* = 0.025), while the opposite was observed when cattle received the high-concentrate diet (Figure 4a). Shannon index, which reflects the diversity of the OTU in samples, presented high-forage diet groups as the most diverse (*p* = 0.013) among treatments, and high-concentrate diet groups as the least (Figure 4b). Moreover, Figure 4c shows the boxplot of OTUs of observed species from the samples. The number of OTUs in both high-forage diet groups were higher (though not significant; *p* = 0.259) than the other groups. The diversity index is used to analyze the temporal and spatial changes in species composition, which reflects whether bacterial communities between groups have differences.

### 3.3. Rumen Epithelial Differentially Expressed Genes between Treatments Assigned as the High-Concentrate (Treated Group) and High-Forage (Control Group) Diets

**Figure 5:**
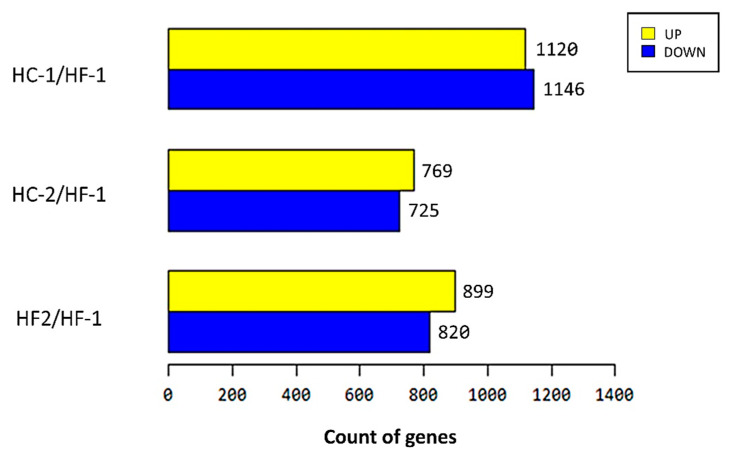
The differentially expressed genes’ (DEGs) up- and down-regulated count among HF-1 (control), HC-1, HC-2, and HF-2 groups. The Y-axis represents up- and down-regulation of DEGs between control vs. treatment groups. The X-axis represents the total number of transcripts (DEGs for each pairwise comparison was selected at FC > = 2). HF-1, high-forage diet (served as control group); HC-1, high-concentrate diet; HC-2, high-concentrate diet; HF-2, high-forage diet.

**Figure 6:**
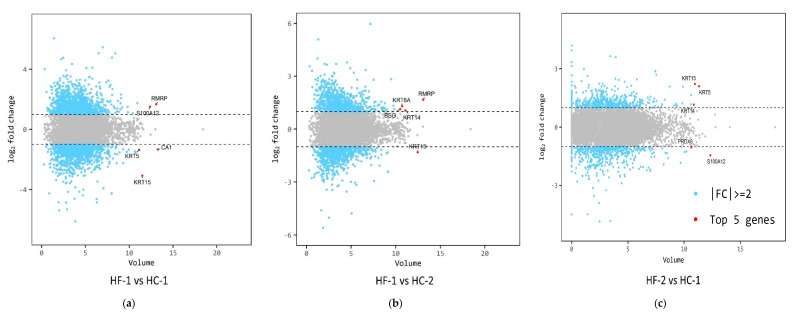
Gene expression volume plot between treatment groups. Genes that showed higher expression difference between HF-1 (control group) and HC-1 (treated group) (**a**). Genes that showed higher expression difference between HF-1 (control group) and HC-2 (treated group) (**b**). HF-2 (control group) and HC-1 (treated group) (**c**). RMRP, RNA component of mitochondrial RNA processing endoribonuclease; S100A12, S100 calcium binding protein A12; CA1, carbonic anhydrase I; KRT5, keratin 5; KRT6A, keratin 6A; KRT14, keratin 14; KRT15, keratin 15; BSG, basigin; PRDX6, peroxiredoxin 6. Red dot: top 5 genes by volume which satisfies, |fc| ≥ 2. FC, fold-change.

**Figure 7:**
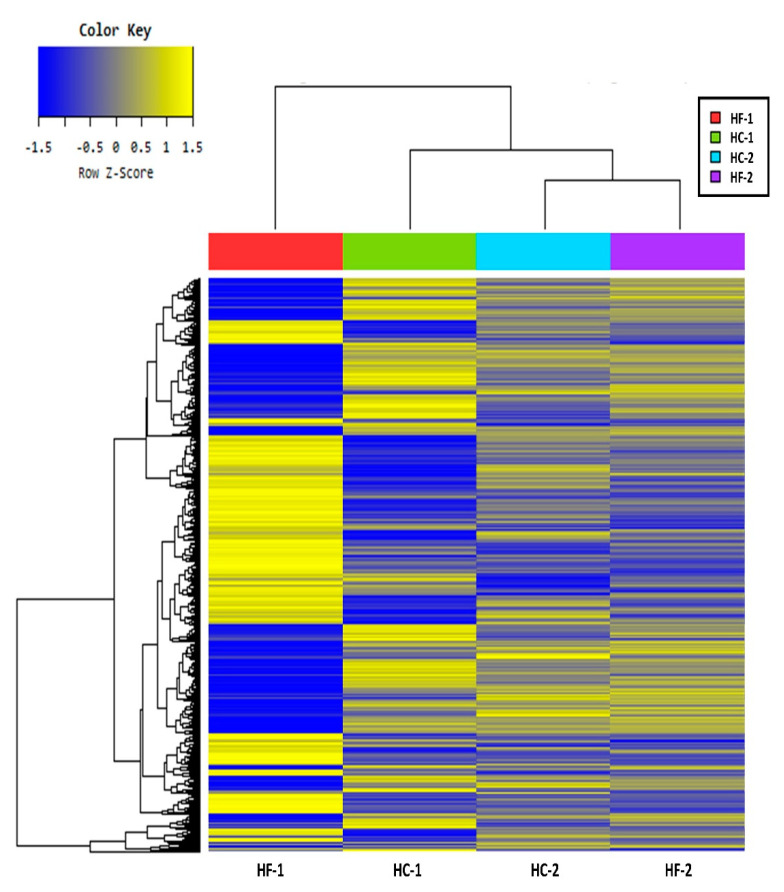
Hierarchical clustering analysis by Euclidean distance and complete linkage showing the similarity of genes and treatments based on normalized value of expression level from the significant list. HF-1, high-forage diet; HC-1, high-concentrate diet; HC-2, high-concentrate diet; HF-2, high-forage diet.

The sequence reads generated per sample ranged from 56,321,752 to 111,981,472 (Table S1). The number of mapped reads also ranged from 65.07% to 88.32%. Based on the mapped data statistics, the high-concentrate diet group had a greater number of reads than the high-forage diet group. There were 2266 (1120 upregulated and 1146 downregulated), 1494 (769 upregulated and 725 downregulated), and 1719 (899 upregulated and 820 downregulated) differentially expressed genes (DEG) when comparing HC-1, HC-2, and HF-2 groups with the HF-1 (control), respectively (Figure 5). The expression volume was defined as the geometric mean of two groups’ expression level. In order to confirm the genes that showed a higher expression difference compared to the control according to expression volume, a volume plot was drawn (Figure 6). It showed a summary of the top expressed genes obtained from the samples between the groups. There were nine top genes obtained after analysis, namely carbonic anhydrase 1, the RNA component of mitochondrial RNA processing endoribonuclease, S100 calcium binding protein A12, keratin 5, keratin 6A, keratin 14, keratin 15, basigin (Ok blood group), and peroxiredoxin 6. Figure 6a shows the top five expressed genes between the diet groups. Carbonic anhydrase I, the RNA component of mitochondrial RNA processing endoribonuclease, and S100 calcium binding protein A12, were highly expressed in HC-1; however, Keratin 15 and 5 were highly expressed in HF-1 (Table S2). Figure 6b shows that the RNA component of the mitochondrial RNA processing endoribonuclease, keratin 14 and 6A, and basigin (Ok blood group) were all highly expressed genes in the concentrate-treated group, except keratin 15, which was found to be highly expressed in the control group (Table S3). Meanwhile, the S100 calcium binding protein A12 and peroxiredoxin 6 were both highly expressed in the treated group, while keratin 1, keratin 14, and keratin 15 were highly expressed in the control group (Table S4, Figure 6c). The results of hierarchical clustering are presented in Figure 7, showing the clustering of genes and samples based on expression level (normalized value) from the significant list. The hierarchical clustering between samples showed that HF-1 was of a different cluster, separating HC-1, as well as HC-2 and HF-2, based on gene expression pattern. This evidently showed that transitioning of diet from high-forage to high-concentrate has an almost completely reversed pattern of expressed genes (HF-1 vs. HC-1). However, during HC-2, a different gene expression pattern was observed compared to the previous period of the same diet. Also, even when the diet was reverted back to high-forage, gene expression was different, which presented a more closely related expression pattern with HC-2.

## 4. Discussion

A sudden decline in ruminal pH is currently one of the major health issues in dairy farming that causes feed intake reduction, digestion problems, and production losses. The prevalence of this mainly affects cattle health and drastically increases management costs. In all ruminants, the rumen is a complex microbial ecosystem and is inhabited by a large density of microbiota, bacteria, anaerobic fungi, archaea, and ciliate protozoa [50]. Rumen microbes have vital roles in the degradation of feedstuffs, and the production of VFAs, lipids, amino acids, and hydrogen, which are essential for the maintenance, growth, and production performance of ruminants [51]. These microbes also supply VFAs, proteins, and vitamins to their ruminant hosts through degrading and fermenting feed materials [17]. A better understanding of the rumen microbiome under extensive feeding conditions is essential due to the complexity of the rumen ecosystem, as the manipulation of ruminal microbiota can improve feed efficiency and optimize rumen function [52,53]. Previous studies have demonstrated that changes in rumen microbial communities are affected by several factors, such as ruminant species, age, health, season, geographical location, feed additives, and diet [18,54,55,56]. Specifically, a diet switch from high forage to high concentrate will definitely cause an enormous change in the rumen bacterial community, which can negatively affect productivity and has the potential to develop metabolic disorders in ruminants [57]. In the present study, we investigated the effect of changing diet on ruminal fermentation characteristics, bacterial community composition, and transcriptome profile of Holstein Friesian cows, which was induced by the transition from a high-forage to a high-concentrate diet, and then returned to a high-forage diet.

Ruminal pH and its daily fluctuations are considered to be a major factor in the occurrence of SARA and the regulation of microbial activity [15,58,59]. Based on the proposed pH thresholds that define ruminal acidosis, it seemed that the Holstein cows in the present study were in SARA condition during the two successive high-concentrate diet periods. A similar result was obtained from the study conducted by Lee et al. [17], wherein the ruminal pH of a Holstein cow drastically decreased upon receiving a high-concentrate diet. The chemical composition of the rumen fluid during the transition from hay to high-concentrate diet can be associated with the development of subacute acidosis [57,60]. A ruminant diet with 35% concentrate reduced the rumen pH below 5.6 for more than 180 min/day, which was an indication of acidosis [15,61,62]. Generally, high-concentrate diets increase both lactic acid producers and utilizers, while decreasing the number of fiber-degrading bacteria. The high level of non-structural carbohydrates from a high-concentrate diet resulted in a drastic decrease in pH of the rumen [63]. Reduction in pH is due to the rapid fermentation of non-structural carbohydrates, and volatile fatty acid accumulation in the rumen [60]. Consequently, reduced pH was also a result of the accumulation of lactic acid in the rumen [17,64]. Moreover, Tajima et al. [64] added that once ruminal pH decreased below 5.6, acid-resistant microbes became dominant in the rumen, which can cause metabolic disorders.

A high concentration of VFA was identified during the high-concentrate diet periods of the present study, which is in accordance with the results of Bevans et al. [65], Sato [66], and Nagata et al. [2]. Lee et al. [17] also stated that a reduction in ruminal pH was directly related to the concentration of total VFA. Previous studies demonstrated that an increase in the VFA concentration, rather than lactate, primarily affected ruminal pH in response to high-concentrate-feeding in cattle [67]. The result of the study by Tajima et al. [57] showed the total VFA concentration tended to increase when cattle were fed a high-concentrate diet. Moreover, Li et al. [15] added that the increase in the concentration of total VFA and the molar proportion of propionate was an indication of a large amount of starch, which usually occurs during the SARA condition. A typical response to an acidotic-like condition in the rumen is changes in the relative proportion of VFA [29,60]. The high concentration of butyrate in the high-concentrate diet period of the current study is also in agreement with the result of Wang et al. [68] and was consistent with that of Ribeiro et al. [69]. Similar to the present study, high propionate concentration in the rumen of cows fed a high-concentrate compared with a high-forage diet has been reported in the literature [70]. Greater propionate production might trigger gluconeogenesis and milk production response [71]. Meanwhile, acetate and butyrate can be converted into each other in the rumen [68]. According to a previous study, 28% of acetate is not absorbed by the rumen in the form of acetate [72] and microorganisms can use this to produce butyrate by acetyl-Coenzyme A transferase and/or butyryl-Coenzyme A transferase [73]. Rumen microbes can promote the metabolism of microorganisms through the energy dissipation process and convert acetate to butyrate continuously during a high-concentrate diet period [73]. Additionally, the present study also supports the claim of Nagata et al. [2], who identified a concurrent increase in the total VFA and NH_3_-N concentrations during the high-concentrate feeding period. Lana et al. [74] reported that forage- and concentrate-fed cattle had various populations of ammonia-producing bacteria in rumen; thus, the optimal ammonia concentration in rumen fluid resulted in the production of microbial protein and the maximum fermentation rate. An NH_3_-N concentration higher than 5 mg/L is the minimum requirement for maximal microbial growth and indicates that a high-concentrate diet can produce a large amount of ruminal microbial crude protein for utilization by the animals [75]. Moreover, the increased NH_3_-N concentration is indicative of an increased rate of proteolysis and amino acid metabolism in the animals [76].

In concordance with many previous studies, the transition of a diet from high forage to high concentrate resulted in various changes in the condition and substrate availabilities in the rumen, which further reduced the richness and diversity of ruminal fluid microbiota [77,78,79,80]. Reports from many studies showed that concentrate-feeding and concentrate-induced SARA alter microbial community structures in the digestive tract [29,30,79]. Ruminant dietary changes contributed significant impacts on rumen bacterial communities [64,81,82]. A previous study demonstrated that dietary forage to concentrate ratio increased from 80:20 to 20:80, and distinctly changed the rumen bacterial population structure [79]. Both studies of Ogata et al. [83,84] presented that low ruminal pH decreased bacterial richness and diversity. During acidosis challenge induced by feeding a concentrate diet, the low ruminal pH could lead to the death and lysis of bacteria, resulting in low relative abundances [84]. The metagenomics survey of bacterial community composition in the present study agreed with previous research showing Firmicutes and Bacteroidetes as the most common phyla in the rumen [29,77,78]. Furthermore, those studies stated that the high-concentrate diet of cattle increased the relative abundance of Firmicutes, while decreasing the abundance of Bacteroidetes. Petri et al. [77] and Henderson et al. [54] also stated that Firmicutes and Bacteroidetes were both identified as the core rumen microbiome. This statement suggests that these two phyla may be less affected by rumen environment changes due to acidic challenges or dietary changes. Excessive grain feeding reduces richness and diversity of rumen microbiota, resulting in a relative abundance reduction of Bacteroidetes and an increase of Firmicutes in the rumen [77,78,85]. Studies have shown that the low proportion of Bacteroidetes during the high-concentrate diet period was due to high acidity in the rumen [66,78]. Meanwhile, Kaoutari et al. [86] concluded that Bacteroidetes was more efficient at degrading structural carbohydrates than Firmicutes. Moreover, the utilization of dietary fiber leads to an increased abundance of Firmicutes rather than Bacteroidetes. Henderson et al. [54] stated that the most dominant taxa in the rumen were the genera *Prevotella*, a well-known degrader of starch, β glycans, protein, pectin, and hemicellulose, and *Ruminococcus*, a cellulose degrader. This statement on the abundance of *Prevotella* was in accordance with the microbiome result of the present study. The ability of this genus to use a variety of substrates allows it to dominate in the rumen under a range of diets [87]. Furthermore, *Prevotella* species are prominent ruminal proteolytic bacteria which produce a variety of extracellular degradative enzymes [88], while *Ruminococcus* are specialized amylolytic bacteria known for the degradation of celluloses in the rumen [89]. *Prevotella ruminicola* appeared to be the predominating species in the present study. It is one of the most numerous groups recovered from the rumen and plays a role in degradation of polysaccharides [90,91,92]. The increase in abundance of *Prevotella marshii* and *Prevotella jejuni* during the high-concentrate diet condition might be due to its cell function as saccharolytic and its ability to ferment glucose [93]. On the other hand, the high prevalence of *Prevotella oralis* under the high-forage condition was likely due to their utilizing function on a wide variety of polysaccharides, and they are thought to be essential contributors to the degradation of xylan [94,95,96,97,98]. Whitehead [99] emphasized that *Prevotella* species in the rumen could contribute to cell wall degradation through synergistic interactions with various species of cellulolytic bacteria. Moreover, the research of Ntougias et al. [100] revealed that *Olivibacter sitiensis* is a xylanolytic bacterium which is involved in the cleavage of *β*-1,4-xylosic bonds in hemicelluloses. This statement supported the abundance of *O. sitiensis* in the present study, which is also observed during a high-forage diet condition. The significant increase in abundance of *Galbibacter mesophilus* and *Erysipelothrix larvae* in the high-forage diet was likely due to its capability to utilize celluloses, which is an important role in regulating the host’s metabolism, thus promoting efficient degradation of polysaccharides [101,102]. Although *Ruminococcus* is a well-known cellulolytic bacterium, several species, such as *R. bromii*, are capable of fermenting starch [103,104]. This may explain the abundance of *R. bromii* in the present study. *Succiniclasticum* are known to ferment succinate and convert it to propionate [51,79,105]. The abundance of *Succiniclasticum ruminis* in the high-concentrate diet periods might explain the high propionate concentration in the rumen. There are also some bacterial species which were found with lower abundance (not presented in figures) that have been reported to exhibit an essential role in pivotal rumen function as rumen homeostasis index due to their contribution in reducing sulfate and its metabolic flexibility [106]. Published studies have shown that this species has the ability to ferment and convert succinate to propionate, which is an essential precursor of glucose in ruminants [105].

In the present study, sufficient information was provided on the differential gene expression pattern in rumen epithelial tissue’s response to the adverse effect of diet transition from a two-week high-forage diet, to a four-week acidosis challenge, back to a two-week high-forage diet. Apart from short-chain fatty acid metabolism, nutrient absorption and transportation are critical functions of the ruminal epithelium [35,107,108,109]. Rumen epithelial structure development is attributed to VFA absorption; thus, the excessive amount of VFA can lead to a reduction in pH that can damage the ruminal epithelium [108,110,111]. Moreover, a high-starch diet in ruminants can cause SARA, which may lead to the destruction of the rumen epithelial tissue in the long term [61,107]. Steele et al. [12] demonstrated that a significant reduction of the total epithelium depth occurred when using a high-concentrate diet-induced acidosis model. In a recent study of Li et al. [35], where cattle were subjected to rumen acidosis by feeding a high-starch diet, the rumen epithelial transcriptome showed a high number of genes that were differentially expressed, impacting biological pathways, specifically genes responsible for cell signaling and morphogenesis. The two assigned groups in the present study had nine major expressed genes, which were confirmed by the high expression difference through the expression volume plot. The high expression of carbonic anhydrase I (CAI) in the high-concentrate-feeding period was in agreement with the results of previous studies [112,113]. They stated that CAI catalyzed the rapid hydration and dehydration of CO_2_ and H_2_CO_3_, respectively. It also played essential roles in physiological systems, such as acid-based balance, respiration, bone resorption, ion transport, ureagenesis, lipogenesis, and gluconeogenesis. Moreover, it secreted parotid saliva containing a HCO_3_^−^-rich alkaline solution, which helped maintain the rumen pH in the range of 6–7 [112]. Meanwhile, the S100 calcium binding protein A12 (S100A12) is an important mediator in various cellular functions which involve apoptosis, cell proliferation, inflammation, and immunity, and is known to be associated with innate immune responses [114,115,116]. This may explain its high expression in the high-concentrate-feeding period, given the fact that these cattle were under SARA conditions, which has been associated with the inflammation of different tissues and organs of dairy cattle [58,110,117]. The high expression volume of the gene responsible for the release of keratin in the high-forage feeding period was supported by the results of previous research [118,119,120]. Keratin is known as the epithelial-specific member of the intermediate filament genes and proteins family, which is responsible for structural support and the regulation of metabolic processes [118]. Fiber-diet-fed ruminants were associated with hard keratins in their epithelium [119]. In a previous report on gene expression, Xiang et al. [120] stated that sheep fed different quality fibrous diets had full-thickness rumen wall tissue due to the keratin produced. The stratified squamous epithelium of the rumen surface was cornified and keratinized to protect it from physical damage caused by ingested plant material [121]. Basigin (BSG), also called CD147 or extracellular matrix metalloproteinase inducer (EMMPRIN), is a transmembrane protein that belongs to the immunoglobulin superfamily and is involved in various pathological and physiological phenomena. It is associated with several proteins, including monocarboxylate transporters (MCTs), which catalyze the proto-linked transport of monocarboxylates such as lactate, pyruvate, and ketone bodies, across the plasma membrane [122]. This protein also facilitates the transport of metabolites from the rumen epithelium to the blood [123,124] and possesses an essential role in rumen pH regulation [125,126,127,128]. These data on BSG support the results of the present study. Furthermore, the high expression volume of peroxiredoxin 6 (PRDX6) in our study was in accordance with previous research [129,130]. Hollmann et al. [129] observed that the expression of PRDX6 was downregulated in high-energy diet-fed animals. Meanwhile, Bondzio et al. [130] described PRDX6 as an important antioxidant enzyme that protects cells against oxidative injury by the reduction of endogenous levels of peroxides. This enzyme might also be involved in maintaining cellular homeostasis in the rumen epithelium during concentrate-supplemented diet adaptation. The comparative analysis of the transcriptome profiles revealed that changing the diet can alter rumen epithelia gene expression [131]. The gene expression pattern in epithelial tissue of the rumen was drastically affected as a result of diet transition. Although, the diet was reverted back to high-forage, the hierarchical clustering of HF-2 based on DEG pattern was more related to the patterns during the acidosis period, which could imply that these harsh changes on the transcriptome of rumen epithelium would require more time to recover to the normal gene expression pattern. Hence, analysis of DEG using RNA sequencing in an extended period of high-forage diet after acidosis challenge is recommended to assess how long would it take for the animals to recover their normal gene expression pattern.

## 5. Conclusions

Ruminal fermentation characteristics, the rumen bacterial community structure, and differentially expressed genes were affected by the changing diet and were induced by transition from a high-forage to a high-concentrate diet. Ruminal pH drastically decreased during the high-concentrate diet period, while a greater increase in concentrations of NH_3_-N and individual and total VFAs were observed. This study was able to confirm the changes in the rumen bacterial community and structure. Among the diets, the high-concentrate diet reduced the richness and diversity of the rumen microbiota. The metagenomics survey on bacterial abundance revealed that Bacteroidetes dominated all the treatments at the phylum level. *Prevotella* abundance significantly differed between the high-forage and high-concentrate diet periods and had a higher relative abundance among the microbial genera. Besides degrading starch, β glycans, protein, pectin, and hemicellulose, they also have the ability to use a variety of substrates, allowing them to dominate the rumen under a range of diets. Meanwhile, there were nine top expressed genes that satisfied the two-fold change based on the expression volume analysis. The differentially expressed genes’ analysis revealed that changing diet can alter gene expression in the rumen epithelia.

## Figures and Tables

**Table 1:** Composition of the Klein grass and concentrate on percentage.

Item ^1^	Klein Grass	Concentrate
Chemical composition (%)		
Crude Protein	12.70	13.03
Crude Fat	1.06	3.84
Crude Ash	8.73	5.77
TDN	60.18	77.00
Calcium	0.43	1.00
Phosphorus	0.14	0.48
NDF	59.57	23.44
ADF	36.09	9.92
Potassium	1.76	0.84
Magnesium	0.48	0.24
Sodium	0.18	0.21

^1^ TDN, total digestible nutrients; NDF, neutral detergent fiber; ADF, acid detergent fiber.

**Table 2:** Rumen fermentation profile of Holstein Friesian cows fed with different diet ratio.

Parameters	Treatments ^1^				SEM ^2^	*p-*Value
	HF-1	HC-1	HC-2	HF-2		
pH	6.87 ^a^	5.58 ^b^	5.41 ^b^	6.89 ^a^	0.098	<0.001
NH_3_-N (mg/dL)	4.88 ^b^	10.99 ^a^	11.37 ^a^	5.04 ^b^	0.896	<0.001
Total VFA (mmol/L)	92.39 ^b^	154.59 ^a^	149.33 ^a^	85.28 ^b^	7.577	<0.001
VFA (mmol/L)						
Acetate	70.12 ^b,c^	95.25 ^a^	86.10 ^a,b^	61.05 ^c^	6.072	0.010
Propionate	14.92 ^b^	39.73 ^a^	40.15 ^a^	15.76 ^b^	5.038	0.001
Butyrate	7.36 ^b^	19.60 ^a^	23.08 ^a^	8.47 ^b^	1.188	<0.001
A/P ratio	4.73 ^a^	2.70 ^b,c^	2.25 ^c^	4.04 ^a,b^	0.437	0.001

^1^ Treatments: HF-1 (high-forage diet); HC-1 (high-concentrate diet); HC-2 (high-concentrate diet); HF-2 (high-forage diet). ^a–c^ Means with different superscripts in a row differ significantly (*p* < 0.05). ^2^ SEM: standard error of the mean. VFA, volatile fatty acid; A/P, acetate to propionate ratio.

## Data Availability

The data presented in this study are available on request from the corresponding author.

## Supplementary Materials

The following are available online at https://www.mdpi.com/2076-2615/11/3/838/s1, Table S1: Mapped cDNA fragments obtained from RNA sequencing, Table S2: Top five expressed genes between HF-1 (control group) and HC-1 (treated group), Table S3: Top five expressed genes between HF-1 (control group) and HC-2 (treated group), Table S4: Top five expressed genes between HF-2 (control group) and HC-1 (treated group), Figure S1: Heatmap showing the relationship of bacterial species on pH values of treatments based on Pearson correlation.
