# Supplementary material for: Diet Transition from High-Forage to High-Concentrate Alters Rumen Bacterial Community Composition, Epithelial Transcriptomes and Ruminal Fermentation Parameters in Dairy Cows
Source: Animals (Basel). 2021 Mar 16;11(3):838. doi: 10.3390/ani11030838 (PMC8002347; doi:10.3390/ani11030838)
Supplement: Supplementary file 1 [file animals-11-00838-s001.zip › Supplementary figure 1.docx]

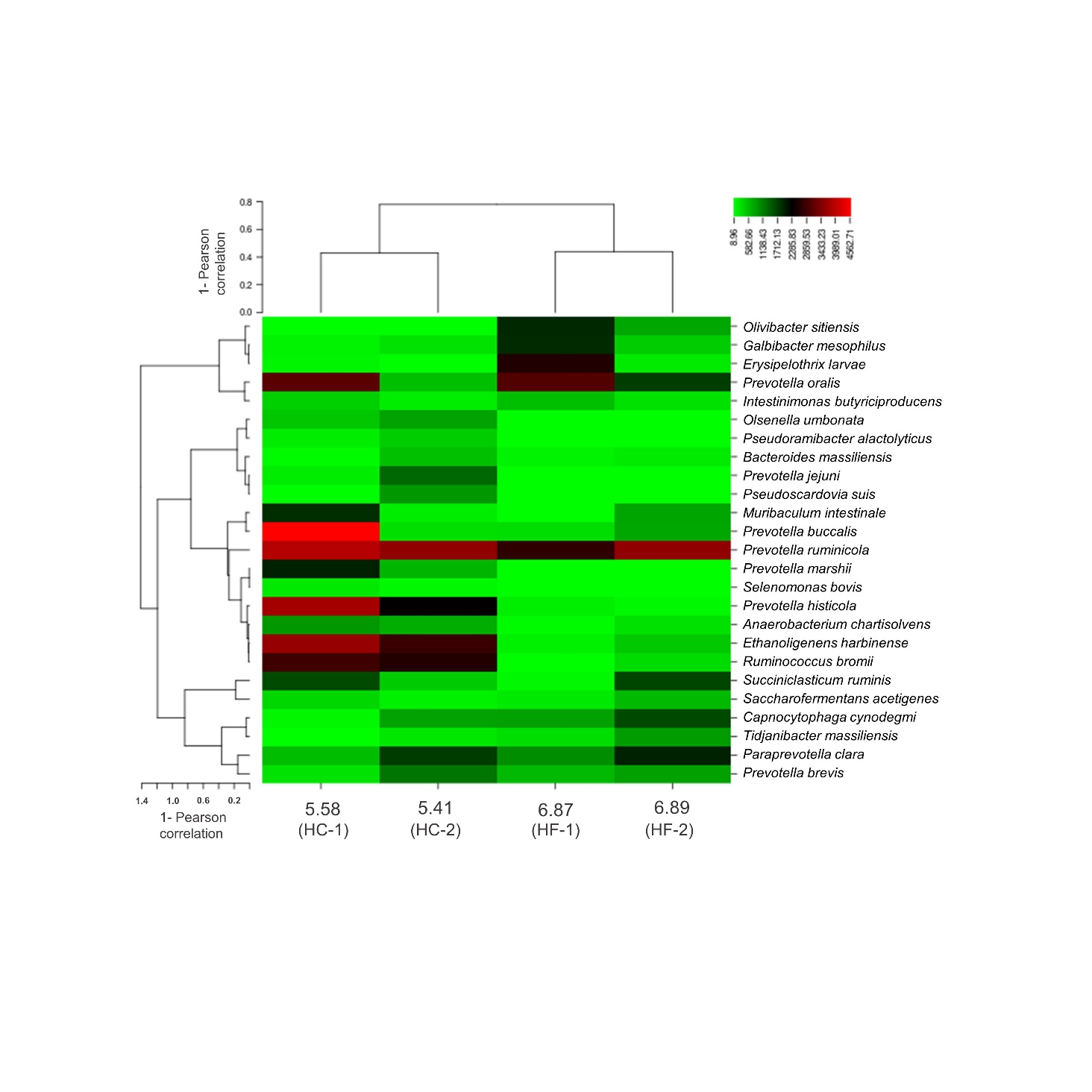


Figure S1. Heatmap showing the relationship of bacterial species on pH values of treatments based on Pearson correlation.
