# Supplementary material for: Diet Transition from High-Forage to High-Concentrate Alters Rumen Bacterial Community Composition, Epithelial Transcriptomes and Ruminal Fermentation Parameters in Dairy Cows
Source: Animals (Basel). 2021 Mar 16;11(3):838. doi: 10.3390/ani11030838 (PMC8002347; doi:10.3390/ani11030838)
Supplement: Supplementary file 1 [file animals-11-00838-s001.zip › Supplementary table.docx]

**Table S1.** Mapped cDNA fragments obtained from RNA sequencing

| Treatments^1^ | Number of processed read | Number of mapped reads (%) | Number of unmapped reads (%) |
| --- | --- | --- | --- |
| HF-1 | 56,321,752 | 36,651,309 (65.07%) | 19,670,443 (34.93%) |
| HC-1 | 111,981,472 | 93,090,399 (83.13%) | 18,891,073 (16.87%) |
| HC-2 | 84,142,334 | 74,311,179 (88.32%) | 9,831,155 (11.68%) |
| HF-2 | 59,052,084 | 45,207,425 (76.56%) | 13,844,659 (23.44%) |

^1^Treatments: HF-1, high-forage diet; HC-1, high-concentrate diet; HC-2, high-concentrate diet; HF-2, high-forage diet.

**Table S2.** Top five expressed genes between HF-1 (control group) and HC-1 (treated group)

| Gene ID | Transcript ID | Gene Symbol | Description | Treatments (FPKM)^1^ | |
| --- | --- | --- | --- | --- | --- |
|  |  |  |  | HF-1 (control group) | HC-1 (treated group) |
| 510801 | NM_001075466 | CA1 | carbonic anhydrase I | 6878.21 | 9394.34 |
| 100126828 | NR_036646 | RMRP | RNA component of mitochondrial RNA processing endoribonuclease | 4161.45 | 16957.65 |
| 282467 | NM_174651 | S100A12 | S100 calcium binding protein A12 | 2684.31 | 10798.57 |
| 507464 | NM_001130747 | KRT15 | keratin 15 | 6575.76 | 1546.33 |
| 281268 | NM_001008663 | KRT5 | keratin 5 | 3140.04 | 1957.57 |

^1^Treatments: HF-1, high-forage diet; HC-1, high-concentrate diet; FPKM, Fragment per Kilobase of transcript per Million mapped Reads.

**Table S3.** Top five expressed genes between HF-1 (control group) and HC-2 (treated group)

| Gene ID | Transcript ID | Gene Symbol | Description | Treatments (FPKM)^1^ | |
| --- | --- | --- | --- | --- | --- |
|  |  |  |  | HF-1 (control group) | HC-2 (treated group) |
| 100126828 | NR_036646 | RMRP | RNA component of mitochondrial RNA processing endoribonuclease | 4161.45 | 20861.13 |
| 507464 | NM_001130747 | KRT15 | keratin 15 | 6575.76 | 2842.17 |
| 404111 | NM_001166575 | KRT14 | keratin 14 | 1422.64 | 2732.10 |
| 614456 | NM_001083510 | KRT6A | keratin 6A | 994.72 | 2231.89 |
| 508716 | NM_001075371 | BSG | basigin (Ok blood group) | 905.26 | 1893.55 |

^1^Treatments: HF-1, high forage diet; HC-2, high concentrate diet; FPKM, Fragment per Kilobase of transcript per Million mapped Reads.

**Table S4.** Top five expressed genes between HF-2 (control group) and HC-1 (treated group)

| Gene ID | Transcript ID | Gene Symbol | Description | Treatments (FPKM)^1^ | |
| --- | --- | --- | --- | --- | --- |
|  |  |  |  | HC-1 (treated group) | HF-2 (control group) |
| 282467 | NM_174651 | S100A12 | S100 calcium binding protein A12 | 10798.57 | 3266.99 |
| 281268 | NM_001008663 | KRT5 | keratin 5 | 1957.57 | 7405.64 |
| 507464 | NM_001130747 | KRT15 | keratin 15 | 1546.33 | 4995.17 |
| 404111 | NM_001166575 | KRT14 | keratin 14 | 1943.91 | 3098.89 |
| 282438 | NM_174643 | PRDX6 | peroxiredoxin 6 | 2989.39 | 922.34 |

^1^Treatments: HF-2, high forage diet; HC-1, high concentrate diet; FPKM, Fragment per Kilobase of transcript per Million mapped Reads.
